# Joint effect of race/ethnicity or location of residence and sex on low density lipoprotein-cholesterol among veterans with type 2 diabetes: a 10-year retrospective cohort study

**DOI:** 10.1186/s12872-020-01730-8

**Published:** 2020-10-15

**Authors:** Erin R. Weeda, Kinfe G. Bishu, Ralph Ward, R. Neal Axon, David J. Taber, Mulugeta Gebregziabher

**Affiliations:** 1 Department of Clinical Pharmacy and Outcome Sciences, College of Pharmacy, Medical University of South Carolina, Charleston, USA; 2 Health Equity and Rural Outreach Innovation Center (HEROIC), Ralph H. Johnson Department of Veterans Affairs Medical Center, Charleston, USA; 3 Department of Public Health Sciences, College of Medicine, Medical University of South Carolina, 135 Cannon Street, Charleston, SC 29425 USA; 4 Department of Medicine, College of Medicine, Medical University of South Carolina, Charleston, USA; 5 Department of Surgery, College of Medicine, Medical University of South Carolina, Charleston, USA

**Keywords:** Diabetes, Gender, Low-density lipoprotein, Race/ethnicity, Rural residence, Veterans

## Abstract

**Background:**

Cardiovascular (CV) disease is the leading cause of death among United States women. Rural residence and ethnic-minority status are individually associated with increased CV mortality. Managing dyslipidemia is important in the prevention of CV mortality. However, the impact of race/ethnicity and location of residence on sex differences in dyslipidemia management is not well understood. Therefore, we sought to understand the joint effects of race/ethnicity and location of residence on lipid management differences between veteran men and women with type 2 diabetes (T2D).

**Methods:**

Veterans Health Administration and Centers for Medicare and Medicaid Services data were used to perform a longitudinal cohort study of veterans with T2D (2007–2016). Mixed effects logistic regression with a random intercept was used to model the association between sex and low-density lipoprotein (LDL) > 100 mg/dL and its interaction with race/ethnicity and location of residence after adjusting for all measured covariates.

**Results:**

When female sex and rural location of residence were both present, they were associated with an antagonistic harmful effect on LDL. Similar antagonistic harmful effects on LDL were observed when the joint effect of female sex and several minority race/ethnicity groups were evaluated. After adjusting for measured covariates, the odds of LDL > 100 mg/dL were higher for urban women (OR = 2.66, 95%CI 2.48–2.85) and rural women (OR = 3.26, 95%CI 2.94–3.62), compared to urban men. The odds of LDL > 100 mg/dL was the highest among non-Hispanic Black (NHB) women (OR = 5.38, 95%CI 4.45–6.51) followed by non-Hispanic White (NHW) women (OR = 2.59, 95%CI 2.44–2.77), and Hispanic women (OR = 2.56, 95%CI 1.79–3.66).

**Conclusion:**

Antagonistic harmful effects on LDL were observed when both female sex and rural location of residence were present. These antagonistic effects on LDL were also present when evaluating the joint effect of female sex and several minority race/ethnicity groups. Disparities were most pronounced in NHB and rural women, who had 5.4 and 3.3 times the odds of elevated LDL versus NHW and urban men after adjusting for important covariates. These striking effect sizes in a population at high cardiovascular risk (i.e., older with T2D) suggest interventions aimed at improving lipid management are needed for individuals falling into one or more groups known to face health disparities.

## Background

Cardiovascular (CV) disease is the leading cause of death among women in the United States (US) [1]. CV morbidity and mortality is substantially increased in patients with type 2 diabetes [2]. In addition to being an independent risk factor for CV disease, diabetes frequently co-exists with conditions known to further elevate CV risk, such as hypertension and dyslipidemia.

Among veterans, women have poorer CV risk factor control than men [3]. For instance, several studies have demonstrated that women are more likely to have elevated low-density lipoprotein cholesterol (LDL) levels than men [3–8]. In a study of 22,888 veterans with CV disease, women were 44% less likely than men to have an LDL < 100 mg/dL (p < 0.001) [8]. Moreover, among approximately 112,000 veterans with diabetes, mean LDL levels were 110 mg/dL in women versus 101 mg/dL in men [7].

Compared with veteran men, a higher proportion of veteran women are racially and ethnically diverse [9]. Race-sex disparities in CV management and outcomes have been described in non-veterans [10–13]. For example, studies including non-veterans have demonstrated that black women are 27% to 34% less likely to achieve LDL goals ranging from 100 mg/dL to 130 mg/dL when compared to white men (p < 0.05 for all comparisons) [10, 13]. However, few studies have evaluated the impact of Hispanic race on sex differences in elevated LDL [14]. Moreover, the impact of race/ethnicity on sex differences in elevated LDL in the veteran population is not well studied.

Among Military recruits, rural residents are overrepresented and rural residence has also been associated with decreased CV risk factor control [15–18]. In a study of 729,822 veterans with diabetes, compared with urban non-Hispanic White (NHW) veterans, rural non-Hispanic Black (NHB) veterans had a 70% higher odds of having LDL levels > 100 mg/dL (p < 0.05) [16]. Yet, the interplay between location of residence and sex and their impact on CV risk factor control is not well studied.

The number of veteran women is increasing, resulting in shifting demographics in the veteran population [3]. Information on disparities in CV risk factor control, including lipid management, is needed. Therefore, we sought to understand the joint effects of race/ethnicity and location of residence on lipid management differences between veteran men and women with type 2 diabetes.

## Methods

### Data

A cohort of veterans with type 2 diabetes was formed via methods established in our previous studies [16, 19, 20] from which details of study design can be obtained. We merged data from the Centers for Medicare and Medicaid Services (CMS) and Veterans Health Administration (VHA) Corporate Data Warehouse (CDW). This VHA dataset was linked with Medicare data (Part A, B, and D) using scrambled social security numbers. The 2007–2016 time period utilized in our study was selected based on inception of Medicare Part D, which was in 2006. This retrospective study was approved by the Medical University of South Carolina (MUSC) institutional review board. No conflicts of interest germane to this manuscript are reported by the authors. The work expresses the authors’ views and not those of the VHA or MUSC.

### Population

We applied selection criteria using methods that we have validated in our previous work [16, 19–21].We first identified individuals cared for at the VHA with type 2 diabetes as indicated by ≥ 2 diagnostic codes (International Classification of Diseases (ICD)-9 codes = 250.x, 357.2, 362.0, and 366.41). The ICD-9 codes had to be present in the 24 months preceding 2002 and again in 2002. Veterans were also required to have hypoglycemic prescriptions in 2002 [19, 21]. We only included veterans ≥ 65 years of age on 01/01/2006, which would make them eligible for Medicare. Those meeting the selection criteria were longitudinally followed from 01/2007 to 12/2016, death or loss to follow-up.

### Covariates and outcomes

Sex (women, men), location of residence (rural, urban) and race/ethnicity served as the primary exposure variables. Race/ethnicity was ascertained from VA and CMS data. The classifications were non-Hispanic Black (NHB), non-Hispanic White (NHW), Hispanic, and Other. Rural Urban Commuting Area (RUCA) codes were used to define location of residence and were derived from zip codes, with urban used for the reference group [22]. Demographics comorbidities [24, 25] and clinical variables were controlled for as described in our previous work [16, 20]. Elevated LDL, defined by two cutoffs (> 100 mg/dL or > 70 mg/dL), was the primary outcome. LDL < 100 mg/dL and < 70 mg/dL was the reference group.

### Analysis

Using one-way ANOVA and *t* tests for continuous variables and chi-square tests for categorical variables we assessed crude associations of clinical characteristics by sex, race/ethnicity and location of residence. After adjusting for all measured covariates, we used mixed effect logistic regression with a random intercept to model the relationship between the primary exposures (sex, location of residence and race/ethnicity) and LDL. While our a-priori hypothesis was to assess joint effects, we also tested the interaction between sex and location of residence as well as sex and race/ethnicity using a likelihood ratio test. Odds ratios (OR) and corresponding 95% confidence intervals (CIs) were estimated after adjustment for clustering and repeated measures via a random intercept. We used the Stata procedure xtlogit to fit the models [26, 27]. As secondary analysis we fitted a linear mixed model with continuous LDL as an outcome using xtmixed. The primary analysis assumes a missing at random mechanism to deal with the 28.8% missing LDL data. A logistic regression model with the missing indicator for LDL as the outcome via xtgee was used to identify covariates most strongly associated with missingness. Moreover, a sensitivity analysis was conducted by fitting the models after imputing the data ten times (multiple imputation with chained equations). These results were consistent with the xtlogit estimates. To assess goodness-of-fit, we used residual analysis. Results of the aforementioned models and sensitivity analyses assessing missingness can be found in Additional file 1: Tables 1–7. Stata ver. 15 (StataCorp. 2017. Stata Statistical Software: Release 15. College Station, TX: StataCorp LLC) was used for all analyses.

**Table 1:** Demographic and clinical characteristics by sex, 2007–2016

Variable	Level	Sex		
		Women	Men	Total
n	No. (%)	9608 (1.3%)	704,604 (98.7%)	714,212 (100.0%)
Age	mean (Std)	76.6 (7.5)	75.9 (6.2)	75.9(6.2)
Sex	Male (%)	–	–	–
Mortality rate	(year 2007)	63.5	67.8	67.8
Race-ethnicity	non-Hispanic white (%)	84.9	82.7	82.7
	non-Hispanic black (%)	9.5	10.0	10.0
	Hispanic (%)	2.5	5.2	5.2
	Other race (%)	3.1	2.1	2.1
Marital status	Married (%)	23.0	60.1	59.6
Disability	> 50% service-related (%)	9.8	17.0	16.9
Location of residence	Rural (%)	29.6	35.3	35.2
Smoking status	Smoker (%)	11.6	14.1	14.1
Number of Elixhauser comorbidities	mean number per group (std)	8.7 (3.4)	8.1 (3.3)	8.1 (3.3)
Number of primary care visits	mean per year (std)	4.7 (4.2)	4.7 (4.1)	4.7 (4.1)
Hemoglobin A1c	Percent ≥ 8% (64 mmol/mol)	9.5	10.1	10.1
	Percent < 8% (64 mmol/mol)	61.6	62.9	62.8
	Missing	28.9	27.0	27.1
ASCVD (%)	Acute coronary syndrome	22.4	24.3	24.3
	Atherosclerotic cerebrovascular disease	23.0	20.7	20.7
	Coronary heart disease	55.4	66.2	66.1
	Peripheral artery disease	38.0	46.4	46.2
Statin use (%)	None	18.5	18.1	18.1
	Low/moderate intensity	69.3	71.2	71.2
	High intensity	4.1	3.5	3.5
	Missing	8.3	7.2	7.2
Dual VA-CMS utilization (%)	> 80% VA utilization	43.6	47.2	47.2
	50–80% VA utilization	5.9	7.4	7.3
	< 50% VA utilization	30.1	32.7	32.7
	Missing	20.4	12.7	12.8
History of comorbidity (%)	Psychiatric disorder	28.0	18.8	19.0
	Depression	45.3	30.9	31.1
LDL ≥ 70 (%)	No	12.6	18.9	18.8
	Yes	57.4	52.3	52.4
	Missing	30.0	28.8	28.8
LDL ≥ 100 (%)	No	40.5	51.2	51.1
	Yes	29.4	20.0	20.1
	Missing	30.0	28.8	28.8

All values are at visit of baseline year (2007)

*ASCVD* atherosclerotic cardiovascular disease, *CMS* Centers for Medicare and Medicaid, *LDL* low-density lipoprotein cholesterol, *VA* Veterans Affairs

## Results

### Demographics

Table 1 displays the characteristics of the cohort (n = 714,212 veterans with type 2 diabetes and 65 years of age or older) stratified by sex. The cohort was predominantly male (98.7%) with a mean age of 75.9 years. The majority were urban residents (64.8%) and NHW (82.7%). Compared with veteran men, veteran women were less likely to be married (60.1% vs. 23.0%), smoke (14.1% vs. 11.6%) and have > 50% service-related disability (17.0% vs. 9.8%). Veteran women were also less likely to have coronary heart disease (CHD; 66.2% vs. 55.4%) but more likely to have atherosclerotic cerebrovascular disease (20.7% vs. 23.0%).

Cohort characteristics stratified by location of residence are displayed in Additional file 1: Table 8. Compared with urban veterans, those living in rural areas were more likely to smoke (13.3% vs. 15.4%) and have ASCVD; including CHD (64.5% vs. 69.1%) and atherosclerotic cerebrovascular disease (20.0% vs. 21.9%). Additional file 1: Table 9 displays cohort characteristics stratified by race/ethnicity. Smoking was most common among NHB veterans (17.6%). While CHD was most common among NHW (68.2%), atherosclerotic cerebrovascular disease was most common among NHB (22.0%).

### Temporal trends in the proportion of veterans with LDL > 100 mg/dL or 70 mg/dL

**Fig. 1:**
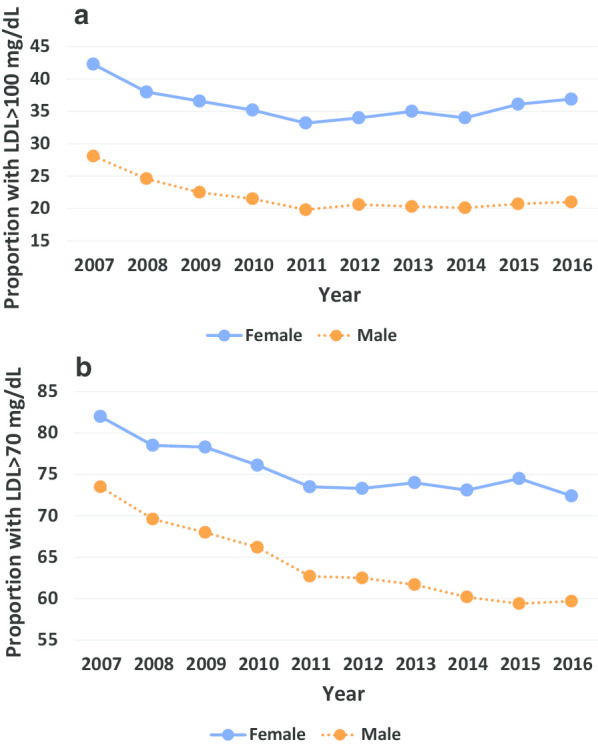
Proportion of patients with LDL cholesterol > 100 mg/dL(A) or > 70 mg/dL(B) over time by sex

Between 2007 and 2016, more veteran women vs. men consistently had elevated LDL levels (> 100 mg/dL and > 70 mg/dL, Fig. 1). Overall, the proportion of veterans with LDL > 100 mg/dL decreased from 42.3 to 33.2% in women and from 28.1 to 19.8% in men over this time period. Similar decreases in the proportion of veteran women and men with an LDL > 70 mg/dL were also observed.

**Fig. 2:**
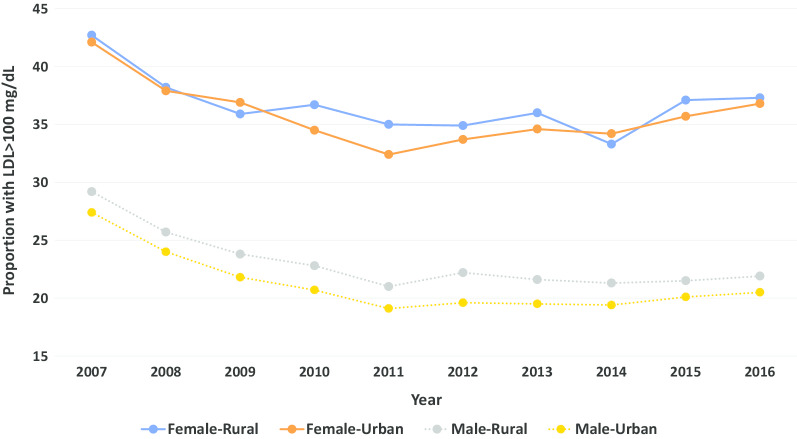
Proportion of patients with LDL cholesterol > 100 mg/dL over time by sex and location

**Fig. 3:**
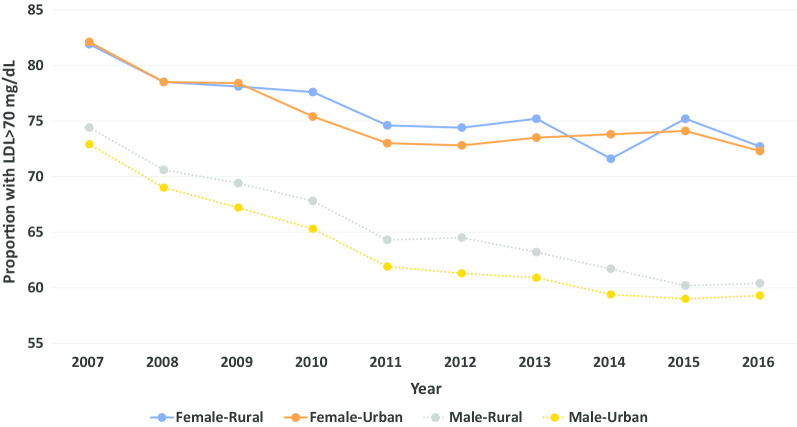
Proportion of patients with LDL cholesterol > 70 mg/dL over time by sex and location

When stratified by sex and location, LDL levels > 100 mg/dL were more common in veteran women than veteran men regardless of location of residence (Fig. 2). The proportion of rural and urban women with LDL levels > 100 mg/dL ranged from 32.4 to 42.1%; while in men these proportions ranged from 19.1 to 29.2%. Similar results were observed for the outcome of LDL > 70 mg/dL (Fig. 3).

**Fig. 4:**
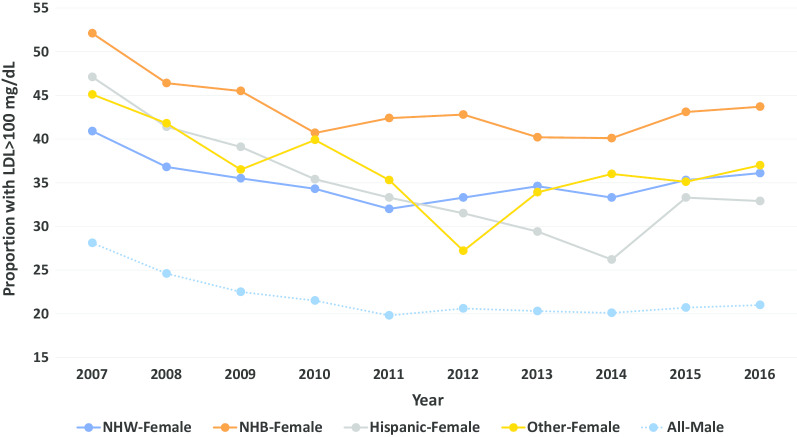
Proportion of patients with LDL cholesterol > 100 mg/dL over time by sex and race/ethnicity

**Fig. 5:**
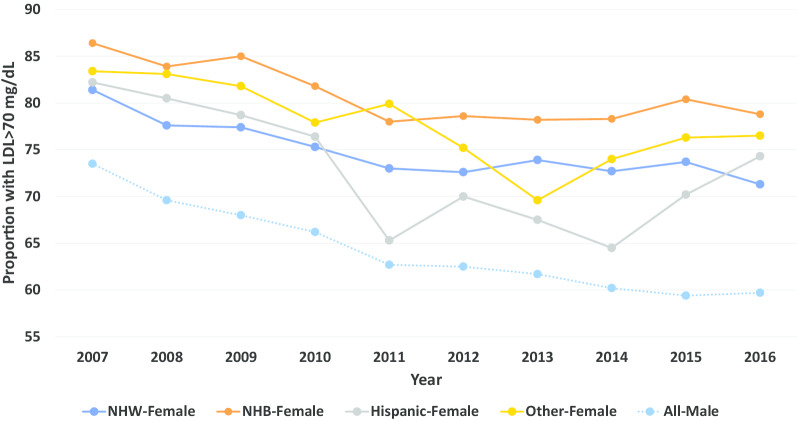
Proportion of patients with LDL cholesterol > 70 mg/dL over time by sex and race/ethnicity

The proportion of veteran women and men with LDL levels > 100 mg/dL between 2007 and 2016 stratified by race and sex is displayed in Fig. 4. When compared with men, women more commonly had LDL levels > 100 mg/dL (range: 19.8 to 28.1% vs. 26.2 to 52.1%, respectively). This relationship was also observed for LDL levels > 70 mg/dL (Fig. 5).

### Sex differences in the likelihood of having elevated LDL levels by location of residence

Table 2 provides models for the joint effect of sex and location of residence. In the base model, male veterans from rural areas had 18% higher odds of having LDL > 100 mg/dL and 17% higher odds of having LDL > 70 mg/dL compared to male veterans from urban areas. Compared to urban male veterans, urban veteran women were more than three times more likely (OR 3.12, 95%CI 2.93, 3.33) to have a LDL > 100 mg/dL and more than 2.4 times as likely (OR 2.41, 95%CI 2.26, 2.58) to have a LDL > 70 mg/dL. Rural women veterans were 3.3 times more likely (OR 3.33, 95%CI 3.02, 3.67) to have a LDL > 100 mg/dL and 2.6 times more likely (OR 2.56, 95%CI 2.31, 2.84) to have a LDL > 70 mg/dL than male veterans from urban areas.

**Table 2:** Sex-location: sequential models for the odds of elevated LDL cholesterol

	LDL ≥ 100		LDL ≥ 70	
	Base model	Full model	Base model	Full model
Variable	n = 673,841	n = 636,985	n = 673,841	n = 636,985
Annual visit (0 to 9)	0.93 (0.93, 0.93)	0.94 (0.94, 0.94)	0.87 (0.87, 0.87)	0.89 (0.89, 0.89)
Sex × location^**^				
Male × urban	Ref	Ref	Ref	Ref
Male × rural	1.18(1.16, 1.19)	1.28 (1.26, 1.30)	1.17 (1.15, 1.18)	1.23 (1.22, 1.25)
Female × urban	3.12 (2.93, 3.33)	2.66 (2.48, 2.85)	2.41 (2.26, 2.58)	2.12 (2.00, 2.28)
Female × rural	3.33(3.02, 3.67)	3.26 (2.94, 3.62)	2.56(2.31, 2.84)	2.53 (2.27, 2.84)
Race-ethnicity				
Non-Hispanic white		Ref		Ref
Non-Hispanic black		1.76 (1.72, 1.80)		1.54 (1.51, 1.58)
Hispanic		1.25 (1.21, 1.29)		1.08 (1.05, 1.11)
Other race		1.13 (1.08, 1.19)		1.00 (0.96, 1.04)
Age (per year)		0.99 (0.99, 1.00)		0.99 (0.99, 0.99)
Marital status				
Married versus unmarried (ref.)		0.86 (0.84, 0.87)		0.94 (0.93, 0.96)
Disability (> 50% service-related)		0.98 (0.96, 1.00)		0.98 (0.96, 1.00)
Smoking status				
Smoker versus non-smoker (ref.)		1.10 (1.08, 1.12)		1.01 (1.00, 1.03)
Number primary care visits (per year)		0.99 (0.99, 0.99)		0.99 (0.99, 0.99)
A1C				
A1C > 8% (64 mmol/mol) versus A1C ≤ 8% (64 mmol/mol) (ref)		1.33 (1.32, 1.35)		1.20 (1.19, 1.22)
ASCVD				
Acute coronary syndrome		1.09 (1.07, 1.11)		0.93 (0.92, 0.95)
Atherosclerotic cerebrovascular disease		1.14 (1.12, 1.16)		1.05 (1.03, 1.07)
Coronary heart dis		0.58 (0.58, 0.59)		0.56 (0.55, 0.57)
Peripheral artery dis		0.93 (0.91, 0.94)		0.89 (0.88, 0.91)
Statins prescribed				
No statin		Ref		Ref
Low/moderate-intensity statin		0.34 (0.32, 0.36)		0.41 (0.38, 0.43)
High-intensity statin		0.29 (0.27, 0.31)		0.29 (0.27, 0.31)
Dual VA-CMS status				
> 80% VA utilization		Ref		Ref
50–80% VA utilization		1.00 (0.98, 1.01)		0.98 (0.96, 0.99)
< 50% VA utilization		0.99 (0.98, 1.00)		0.93 (0.92, 0.94)
History of comorbidity				
Psychiatric disorder		1.16 (1.14, 1.18)		1.06 (1.04, 1.07)
Depression		1.17 (1.15, 1.19)		1.08 (1.07, 1.10)
Inter cluster correlation	0.57 (0.57, 0.57)	0.56 (0.56, 0.57)	0.56 (0.56, 0.56)	0.56 (0.56, 0.56)

LDL measured in mg/dL. Data measured between 2007 and 2016. Odds ratios (95% confidence intervals) from logistic random-intercept models

*ASCVD* atherosclerotic cardiovascular disease, *CMS* Centers for Medicare and Medicaid, *LDL* low-density lipoprotein cholesterol, *VA* Veterans Affairs

^**^p < 0.001

In the fully adjusted model, the odds of LDL > 100md/dL were 2.7 times higher in urban women (OR 2.66, 95%CI 2.48, 2.85) and 3.3 times (OR 3.26, 95% CI 2.94, 3.62) higher in rural women compared to urban men. The results for the outcome of LDL > 70 mg/dL were similar, with the odds increased among urban women (OR 2.12, 95% CI 2.00–2.28) and rural women (OR 2.53, 95% CI 2.27–2.84) compared to urban men.

### Sex differences in the likelihood of having an elevated LDL by race/ethnicity

Table 3 provides sequential models for the joint effect of sex and race/ethnicity. In the base model, NHW female veterans were three times (OR 3.01, 95% CI 2.84, 3.19), NHB male were 1.80 times (OR 1.80, 95% CI 1.76, 1.84), NHB women were nearly six times (OR 5.97, 95% CI 5.05, 7.06), Hispanic male were 1.3 times (OR 1.31, 95% CI 1.27, 1.34), Hispanic women were 3.7 times (OR 3.72, 95% CI 2.68, 5.16), other race male were 1.2 times (OR 1.15, 95% CI 1.10, 1.21) and other race female were 3.3 times (OR 3.34, 95% CI 2.45, 4.56) more likely to have LDL levels > 100 mg/dL compared to NHW male. Similarly, NHW females were 2.3 times (OR 2.29, 95% CI 2.16, 2.44), NHB male were 1.6 times (OR 1.57, 95% CI 1.53, 1.60), NHB women were 4.4 times (OR 4.41, 95% CI 3.66, 4.31), Hispanic male were 1.2 times (OR 1.15, 1.12, 1.18), Hispanic women were 2.3 times (OR 2.26, 1.60, 3.18), and other race women were 2.9 times (OR 2.85, 2.04, 3.97) more likely to have a LDL > 70 mg/dL compared to NHW male; with no difference observed for the other race male cohort.

**Table 3:** Sex-race: sequential models for the odds of elevated LDL cholesterol

	LDL ≥ 100		LDL ≥ 70	
	Base model	Full model	Base model	Full model
Variable	n = 673,841	n = 636,985	n = 673,841	n = 636,985
Annual visit (0 to 9)	0.93 (0.93, 0.93)	0.94 (0.94, 0.94)	0.87 (0.87, 0.87)	0.89 (0.89, 0.89)
Sex × race^**^				
Non-Hispanic white × male	Ref	Ref	Ref	Ref
Non-Hispanic white × female	3.01 (2.84, 3.19)	2.59 (2.44, 2.77)	2.29 (2.16, 2.44)	2.06 (1.93, 2.20)
Non-Hispanic black × male	1.80 (1.76, 1.84)	1.76 (1.72, 1.80)	1.57 (1.53, 1.60)	1.54 (1.51, 1.58)
Non-Hispanic black × female	5.97 (5.05, 7.06)	5.38 (4.45, 6.51)	4.41 (3.66, 4.31)	3.93 (3.18, 4.87)
Hispanic × male	1.31 (1.27, 1.34)	1.25 (1.21, 1.29)	1.15 (1.12, 1.18)	1.08 (1.05, 1.11)
Hispanic × female	3.72 (2.68, 5.16)	2.56 (1.79, 3.66)	2.26 (1.60, 3.18)	1.83 (1.26, 2.64)
Other race × male	1.15 (1.10, 1.21)	1.13 (1.08, 1.19)	1.02 (0.98, 1.06)	1.00 (0.95, 1.04)
Other race × female	3.34 (2.45, 4.56)	2.98 (2.11, 4.22)	2.85 (2.04, 3.97)	2.58 (1.77, 3.76)
Location of residence				
Urban		Ref		Ref
Rural		1.28 (1.26, 1.30)		1.23 (1.22, 1.25)
Age (per year)		0.99 (0.99, 1.00)		0.99 (0.99, 0.99)
Marital status				
Married versus unmarried (ref.)		0.86 (0.84, 0.87)		0.94 (0.93, 0.96)
Disability (> 50% service-related)		0.98 (0.96, 1.00)		0.98 (0.96, 0.99)
Smoking status				
Smoker versus non-smoker (ref.)		1.10 (1.08, 1.12)		1.01 (0.99, 1.03)
Number primary care visits (per year)		0.99 (0.99, 0.99)		0.99 (0.99, 0.99)
A1C				
A1C > 8% (64 mmol/mol) versus A1C ≤ 8% (64 mmol/mol) (ref)		1.33 (1.32, 1.35)		1.20 (1.19, 1.22)
ASCVD				
Acute coronary syndr		1.09 (1.07, 1.11)		0.93 (0.92, 0.95)
Atheroscl. cerebro. dis		1.14 (1.12, 1.19)		1.05 (1.03, 1.07)
Coronary heart dis		0.59 (0.58, 0.60)		0.56 (0.55, 0.57)
Peripheral artery dis		0.93 (0.92, 0.93)		0.89 (0.88, 0.90)
Statins prescribed				
No statin		Ref		Ref
Low/moderate-intensity statin		0.34 (0.32, 0.36)		0.41 (0.38, 0.43)
High-intensity statin		0.29 (0.27, 0.31)		0.29 (0.27, 0.31)
Dual VA-CMS status				
> 80% VA utilization		Ref		Ref
50–80% VA utilization		1.00 (0.99, 1.02)		0.98 (0.96, 0.99)
< 50% VA utilization		0.99 (0.98, 1.00)		0.93 (0.92, 0.94)
History of comorbidity				
Psychiatric disorder		1.16 (1.14, 1.18)		1.06 (1.03, 1.07)
Depression		1.17 (1.15, 1.19)		1.08 (1.07, 1.10)
Inter cluster correlation	0.56 (0.56, 0.56)	0.56 (0.56, 0.56)	0.56 (0.56, 0.56)	0.56 (0.56, 0.56)

LDL measured in mg/dL. Data measured between 2007 and 2016. Odds ratios (95% confidence intervals) from logistic random-intercept models

*ASCVD* atherosclerotic cardiovascular disease, *CMS* Centers for Medicare and Medicaid, *LDL* low-density lipoprotein cholesterol, *VA* Veterans Affairs

**p < 0.001

In the fully adjusted model, the odds of LDL > 100 mg/dL was 2.6 times higher in NHW women (OR 2.59, 95% CI 2.44, 2.77) than NHW men. Similarly, NHB women had 5.4 times higher odds of LDL > 100 mg/dL compared to NHW men (OR 5.38, 95% CI 4.45, 6.51). Hispanic women and women in the other race cohort had 2.6 (OR 2.56, 95% CI 1.79, 3.66) and 3 times (2.98, 95% CI 2.11, 4.22) the odds of LDL > 100 mg/dL compared to NHW men. The results were similar for the odds of having LDL > 70 mg/dL, with higher odds among NHW women (OR 2.06, 95% CI 1.93, 2.20), NHB women (OR 3.93, 95% CI 3.18, 4.87), Hispanic women (1.83, 95% CI 1.26–2.64) and women in the other race cohort (OR 2.58, 95% CI 1.77, 3.76) compared with NHW men.

The interaction between sex and location as well as sex and race/ethnicity were statistically significant (p < 0.001; Tables 2 and 3). Moreover, analyses of LDL as continuous variable using a linear mixed model were consistent with the results in Tables 2 and 3 for the dichotomized LDL outcome (see supplementary information: Additional files 1: Tables 1 and 2).

## Discussion

In this study of roughly 700,000 older veterans with type 2 diabetes, women were substantially more likely to have elevated LDL levels, when compared to men. Harmful antagonistic effects on LDL were observed when both female sex and rural location of residence were present. These antagonistic effects on LDL were also present when evaluating the joint effect of female sex and several minority race/ethnicity groups. The p-value for the interaction between sex and location as well as sex and race/ethnicity was < 0.001, indicating that the observed antagonistic effects are statistically significant. The adjusted odds of an LDL > 100 mg/dL were 3.3 times higher in women living in rural areas compared to men living in urban areas. The magnitude of the sex differences was more pronounced by race. NHB women had 5.4 times higher odds of a LDL > 100 mg/dL compared to NHW men. The striking magnitude of these effect sizes coupled with the fact that we evaluated a population at high-CV risk (i.e., diabetes, older, > 60% with CHD) suggests that improved strategies for lipid management in veteran women are needed.

Although the interplay between location of residence and sex and their impact on CV risk factor control is not well studied, our findings are consistent with several previous studies reporting disparities in lipid levels among women [3–8, 10–13]. These disparities appear to be most pronounced in black women. In a study of 3484 older patients with hypertension and dyslipidemia, white women were 25% and black women were 34% less likely to have controlled LDL levels (defined as < 100 mg/dL or < 130 mg/dL depending on CV risk) compared with white men (p < 0.05) [13]. This was observed even though black women were receiving the highest potency lipid lowering therapy in the study. Similarly, in a study of ~ 4000 patients over 45 years of age with diabetes, white women were 11% and black women were 27% less likely than white men to have an LDL < 100 mg/dL (p < 0.001) [10]. Moreover, among 5018 patients over 67 years of age, white women were more likely to have controlled LDL (defined as < 100 mg/dL or < 130 mg/dL depending on CV risk) than black women (prevalence ratio = 1.25; 95%CI = 1.08 to 1.46) [12]. Taken together these studies, which are also consistent with the data presented in this study, demonstrates that race has a negative impact on sex disparities in lipid management. Given that two severe consequences of dyslipidemia, death from stroke and myocardial infarction, occur more frequently in black women than white women, this is an especially concerning observation [1].

When female sex and rural location of residence were both present, they were associated with an antagonistic harmful effect on LDL in our analyses. This is similar to findings from an analysis of approximately 700,000 veterans by Brown and colleagues, where rurality had a negative effect on racial disparities in LDL control [16]. When cohorts were defined by race and location of residence, the odds of having a LDL > 100 mg/dL were highest among NHB-rural veterans. These findings are consistent with observations of higher CV mortality among rural residents, which has been attributed to poorer CV risk factor control [16–18]. Adults living in rural areas are less likely than those in urban areas to meet recommendations for aerobic exercise through leisure-time activities (1). Moreover, rural residence is associated with higher rates of smoking, less access to nutritious food and a lower likelihood of receiving preventative care [18, 28].

The antagonistic harmful effect on LDL observed when both rural residence and female sex were present in our analysis is notable for several other reasons. Urban female veterans were 2.66 times more likely than urban males to have LDL ≥ 100 mg/dL and 2.12 times more likely to have LDL ≥ 70 mg/dL. By contrast, rural female veterans were 3.23 and 2.53 times more likely to have elevated LDL at each cut point. Moreover, while LDL cholesterol levels decreased in all groups over our 10 year observation period, female/male disparities persisted in each group over time. In our study, both men and women veterans appear to have had similar and relatively high utilization of primary care clinic visits, with a mean of 4.7 visits per year in each group. However, some prior studies have demonstrated a higher reliance on Medicare for primary care by rural veterans, and other studies have shown VA care to be associated with higher rates of LDL cholesterol control, hemoglobin A1c control, and blood pressure control, compared to Medicare Advantage [29, 30]. These findings are relevant in light of recent VA initiatives to improve access to care for veterans including the Veterans Choice and Accountability Act, a federal law passed in 2014 which significantly expanded utilization of non-VA care among veterans [31]. More recently, the Maintaining Internal Systems and Strengthening Integrated Outside Networks Act of 2018 (MISSION Act) has merged and simplified existing community care programs in VA and expanded circumstances in which non-VA community care can be authorized [32]. Our findings highlight some potential complexities in ensuring new VA access to care initiatives achieve desired effects on health outcomes while reducing disparities in care.

We hypothesize that the disparities observed in our study are likely due to several factors. Veteran women often have less social support than veteran men and social support is associated with greater confidence in one’s ability to control cholesterol and improved CV outcomes [33, 34]. Previous studies have demonstrated that veteran women are more likely to be unmarried, live alone, and report having a lack of help to track their medications or take them to the healthcare appointments [34, 35]. This appeared to be true in our analysis as only 23% of veteran women were married, as compared with 60% of veteran men. Studies have also demonstrated that compared with men, women are less likely to be offered and prescribed lipid lowering therapy [36, 37]. In a prospective study of 5693 patients that qualified for statin treatment, approximately 60% of the 1511 patients that were not on statins, reported never being offered this therapy [37]. Women were 22% more likely than men to report never being offered a statin (p < 0.001). Other factors associated with never being offered statin therapy included black race (relative risk [RR] = 1.48) and the lack of insurance (RR = 1.38; p < 0.001 for both). Moreover, in a study assessing roughly 42,000 provider responses to electronic health record clinical reminders for an elevated LDL (defined as > 100 mg/dL) in veterans with CV disease and diabetes, providers were 25% less likely to order or adjust lipid lowering medication regimens for women than men (p < 0.05) [36]. Women were also 71% more likely to refuse medication therapy (p < 0.05). The authors offered several explanations for their findings. First, women veterans may be more likely to present with competing health issues (e.g., chronic pain, depression), which could shift the discussion away from CV prevention. Further, clinicians may inappropriately conclude that women are at lower CV risk and thus may prioritize other preventative care (e.g., sex-specific preventative care) over CV prevention. Our findings suggest the impact of the aforementioned factors leading to sex disparities is greater when combined with factors known to result in racial disparities (e.g., implicit or explicit bias) and disparities among rural residents (e.g., decreased access to nutritious food or preventative care) [16, 18, 28]. Veterans that fall into more than one group known to face disparities appear to be most vulnerable to having elevated LDL levels and future interventions aimed at improving lipid management should be specifically designed for these high-risk populations.

Our study has several limitations worthy of discussion. First, we could not evaluate several factors known to influence outcomes; most importantly socioeconomic status including income and education. If socioeconomic status was evaluated in this analysis, it would likely also demonstrate an antagonistic harmful effect on LDL. Second, aside from marital status, we were not able to evaluate other markers of social support (e.g., living alone, having someone else present at medical visits, having help keeping track of medications), which are known to improve outcomes among veterans with CV disease [33, 34]. Third, the sample size for females is much smaller than males. However, this is reflective of the true sex distribution of the US veterans, although it may lead to limited external application of these results outside of the VA system. Fourth, we only included veterans with diabetes and although those with diabetes are at high-risk of cardiovascular events related to dyslipidemia, this may decrease applicability to patients without diabetes. Lastly, our results may not be applicable to younger patients, as we only included those 65 years of age or older. This is important to note as the proportion of veterans less than 65 years of age that are women is increasing [3]. Nonetheless, the most concerning consequences of dyslipidemia, myocardial infarction and stroke, occur more frequently in women over 65 years of age [1].

## Conclusion

Among approximately 700,000 veterans with diabetes, we observed sex disparities in lipid management. We found an antagonistic harmful effect on LDL when both female sex and rural location of residence were present. These antagonistic effects on LDL were also present when evaluating the joint effect of female sex and several minority race/ethnicity groups. Consistent across all analyses, veteran women were more than twice as likely as veteran men to have elevated LDL levels (either > 100 mg/dL or > 70 mg/dL). Disparities were most pronounced in NHB-women, who had 5.4 times the odds of LDL levels > 100 mg/dL versus NHW-men after covariate adjustment, and rural women, who had 3.3 times the odds of LDL levels > 100 mg/dL versus urban men after covariate adjustment. These striking effect sizes are especially concerning considering we included a population at high CV risk (i.e., diabetes, older, > 60% had CHD). As patients falling into more than one group known to face disparities appear to be most vulnerable to elevated LDL levels, interventions aimed at improving lipid management should be specifically developed for these individuals.

## Supplementary information

**Additional file 1**. Additional file contains additionals tables 1 to 9.

## Data Availability

The datasets generated and/or analyzed during the current study are not publicly available. Patient records were anonymized and de-identified prior to analysis. Department of Veterans Affairs policy and U.S. federal law prohibit disclosure of Veteran information outside the U.S. Veterans Health Administration. However, investigators interested in obtaining data to replicate our analysis can learn more at the VA Information Resource Center (VIReC) at (https://www.virec.research.va.gov/Index.asp).

## Abbreviations

CV: Cardiovascular
US: United States
LDL: Low-density lipoprotein cholesterol
NHW: Non-Hispanic White
NHB: Non-Hispanic Black
VHA: Veterans Health Administration
CDW: Corporate Data Warehouse
CMS: Centers for Medicare and Medicaid Services
VistA: Veterans Health Information Systems and Technology Architecture
ICD: International Classification of Diseases
RUCA: Rural Urban Commuting Area
ASCVD: Atherosclerotic cardiovascular disease
CHD: Coronary heart disease

## Permissions for data

Permissions to access the Veterans Health Administration (VHA) Corporate Data Warehouse (CDW) and Centers for Medicare and Medicaid (CMS) data were obtained from the Veteran Health Administration, with approval granted by the Research and Development Committee at the Ralph H. Johnson Veteran Affairs Medical Center.
