# Supplementary material for: Joint effect of race/ethnicity or location of residence and sex on low density lipoprotein-cholesterol among veterans with type 2 diabetes: a 10-year retrospective cohort study
Source: BMC Cardiovasc Disord. 2020 Oct 15;20:449. doi: 10.1186/s12872-020-01730-8 (PMC7558630; doi:10.1186/s12872-020-01730-8)
Supplement: Supplementary file 1 — Additional file 1. Additional file contains additionals tables 1 to 9. [file 12872_2020_1730_MOESM1_ESM.docx]

**Additional files**

**Additional File 1: Sex-Location: Linear mixed effect models of elevated LDL cholesterol^*^**

|  | **Full model** |
| --- | --- |
| **Variable** | n=636,943 |
| Annual Visit (0 to 9) | -0.79 (-0.80, -0.78) |
| **Sex * location** |  |
| male*urban | Ref |
| male*rural | 2.13 (2.00, 2.25) |
| female*urban | 10.77 (10.15, 11.39) |
| female*rural | 12.7 (11.75, 13.62) |
| **Race-ethnicity** |  |
| non-Hispanic white | Ref |
| non-Hispanic black | 5.20 (5.01, 5.40) |
| Hispanic | 1.02 (0.76, 1.27) |
| Other race | 0.78 (0.38, 1.17) |
| Age (per year) | -0.07 (-0.08, -0.06) |
| **Marital status** |  |
| Married vs unmarried (ref.) | -1.05(-1.16, -0.93) |
| Disability (>50% service-related) | -0.33 (-0.48, -0.18) |
| **Smoking Status** |  |
| Smoker vs non-smoker (ref.) | 0.44 (0.28, 0.60) |
| Number primary care visits (per year) | -0.08 (-0.09, -0.08) |
| **A1C** |  |
| A1C >8% (64 mmol/mol) vs A1C ≤8% (64 mmol/mol) (ref) | 2.40 (2.32, 2.48) |
| **ASCVD** |  |
| Acute coronary syndrome | 0.28(0.14,0.43) |
| Atherosclerotic   cerebrovascular disease | 1.16 (0.97, 1.26) |
| Coronary heart dis. | -4.85 (-4.98, -4.72) |
| Peripheral artery dis. | -0.91 (-1.02, -0.79) |
| **Statins prescribed** |  |
| No statin | Ref |
| Low/Moderate-intensity statin | -8.98 (-9.38, -8.58) |
| High-intensity statin | -13.10 (-13.50, -12.69) |
| **Dual VA-CMS status** |  |
| >80% VA utilization | Ref |
| 50-80% VA utilization | -0.22 (-0.31, -0.13) |
| < 50% VA utilization | -0.30 (-0.38, -0.22) |
| **History of comorbidity** |  |
| Psychiatric disorder | 0.94 (0.78, 1.10) |
| Depression | 1.06 (0.93, 1.20) |

ASCVD=atherosclerotic cardiovascular disease; CMS=Centers for Medicare and Medicaid; LDL=low-density lipoprotein cholesterol VA=Veterans Affairs

*LDL measured in mg/dL. Data measured between 2007-2016. Linear Mixed Effect Coef. (95% Confidence Intervals) random-intercept models.

**Additional File 2: Sex-Race: Linear mixed effect models of elevated LDL cholesterol^*^**

|  | **Full model** |
| --- | --- |
| **Variable** | n=636,943 |
| Annual Visit (0 to 9) | -0.79(-0.80,-0.78) |
| Non-Hispanic white*male | Ref |
| Non-Hispanic white *female | 10.42(9.86,10.98) |
| Non-Hispanic black*male | 5.17(4.97,5.36) |
| Non-Hispanic black*female | 18.67(16.96,20.36) |
| Hispanic*male | 1.02(0.76,1.28) |
| Hispanic*female | 10.43(7.26,13.60) |
| Other race*male | 0.76(0.36,1.16) |
| Other race*female | 12.38(9.28,15.48) |
| **Location of residence** |  |
| Urban | Ref |
| Rural | 2.13 (2.01,2.25) |
| Age (per year) | -0.07(-0.08,-0.06) |
| **Marital status** |  |
| Married vs unmarried (ref.) | -1.05(-1.16, -0.93) |
| Disability (>50% service-related) | -0.33 (-0.48, -0.18) |
| **Smoking Status** |  |
| Smoker vs non-smoker (ref.) | 0.44 (0.28, 0.60) |
| Number primary care visits (per year) | -0.08 (-0.09, -0.08) |
| **A1C** |  |
| A1C >8% (64 mmol/mol) vs A1C ≤8% (64 mmol/mol) (ref) | 2.40 (2.32, 2.48) |
| **ASCVD** |  |
| Acute coronary syndrome | 0.28(0.14,0.43) |
| Atherosclerotic   cerebrovascular disease | 1.11 (0.97, 1.26) |
| Coronary heart dis. | -4.85 (-4.98, -4.72) |
| Peripheral artery dis. | -0.91 (-1.02, -0.79) |
| **Statins prescribed** |  |
| No statin | Ref |
| Low/Moderate-intensity statin | -8.98 (-9.38, -8.58) |
| High-intensity statin | -13.10 (-13.50, -12.69) |
| **Dual VA-CMS status** |  |
| >80% VA utilization | Ref |
| 50-80% VA utilization | -0.22 (-0.31, -0.13) |
| < 50% VA utilization | -0.30 (-0.38, -0.22) |
| **History of comorbidity** |  |
| Psychiatric disorder | 0.94 (0.78, 1.10) |
| Depression | 1.06 (0.93, 1.20) |

ASCVD=atherosclerotic cardiovascular disease; CMS=Centers for Medicare and Medicaid; LDL=low-density lipoprotein cholesterol VA=Veterans Affairs

*LDL measured in mg/dL. Data measured between 2007-2016. Linear Mixed Effect Coef. (95% Confidence Intervals) random-intercept models.

**Additional File 3: Sex-Location: Sequential models for the odds of elevated LDL cholesterol (in mg/dL), 2007-2016.**

| **Odds Ratios (95% Confidence Intervals) for Xtlogit (Logistic Random-Intercept Models)** | | |
| --- | --- | --- |
|  | **LDL≥ 100** | **LDL≥ 70** |
|  | **Full model (No history of comorbidity)** | **Full Model (No history of comorbidity)** |
| **Variable** | n=636,985 | n=636,985 |
| Annual Visit (0 to 9) | 0.94 (0.94,0.94) | 0.89 (0.88,0.89) |
| **Sex * location** |  |  |
| male*urban | ref | Ref |
| male*rural | 1.28 (1.26, 1.30) | 1.23 (1.22, 1.25) |
| female*urban | 2.76 (2.57, 2.96) | 2.16 (2.00, 2.32) |
| female*rural | 3.38 (3.04, 3.75) | 2.58 (2.31, 2.88) |
| **Race-ethnicity** |  |  |
| non-Hispanic white | ref | Ref |
| non-Hispanic black | 1.75(1.71,1.79) | 1.53(1.51,1.57) |
| Hispanic | 1.26(1.23,1.30) | 1.09(1.05,1.12) |
| Other race | 1.13(1.08,1.18) | 1.00(0.95,1.04) |
| Age (per year) | 0.99(0.99, 1.00) | 0.99(0.99,0.99) |
| **Marital status** |  |  |
| Married vs unmarried (ref.) | 0.84(0.83,0.85) | 0.94(0.92,0.95) |
| Disability (>50% service-related) | 1.02(1.00,1.04) | 1.00(0.98,1.01) |
| **Smoking Status** |  |  |
| Smoker vs non-smoker (ref.) | 1.11(1.09,1.13) | 1.01(1.00,1.03) |
| Number primary care visits (per year) | 0.99(0.99,0.99) | 0.99(0.99,0.99) |
| **A1C** |  |  |
| A1C >8% (64 mmol/mol) vs A1C ≤8% (64 mmol/mol) (ref) | 1.34(1.32,1.35) | 1.21(1.19,1.22) |
| **ASCVD** |  |  |
| Acute coronary syndr. | 1.10(1.08,1.12) | 0.94(0.92,0.95) |
| Atheroscl. cerebro. dis. | 1.17(1.15,1.19)) | 1.06(1.04,1.08) |
| Coronary heart dis. | 0.59(0.58,0.60) | 0.56(0.55,0.57) |
| Peripheral artery dis. | 0.93(0.92,0.95) | 0.90(0.89,0.91) |
| **Statins prescribed** |  |  |
| No statins | ref | ref |
| Low/moderate-intensity statin | 0.34(0.32,0.36) | 0.41(0.38,0.44) |
| High-intensity statin | 0.29(0.28,0.31) | 0.29(0.27,0.31) |
| **Dual VA-CMS status** |  |  |
| >80% VA utilization | ref | ref |
| 50-80% VA utilization | 1.00 (0.99,1.02)) | 0.98(0.96,0.99) |
| < 50% VA utilization | 0.99 (0.98,1.00 | 0.93(0.92,0.94) |
| **History of comorbidity** |  |  |
| Psychiatric disorder |  |  |
| Depression |  |  |
| Inter Cluster Correlation | 0.56(0.56,0.57) | 0.56(0.56,0.56) |

**Additional File 4: Sex-Race: Sequential models for the odds of elevated LDL cholesterol (in mg/dL), 2007-2016.**

| **Odds Ratios (95% Confidence Intervals) for Xtlogit (Logistic Random-Intercept Models)** | | |
| --- | --- | --- |
|  | **LDL≥100** | **LDL≥70** |
|  | **Full model (No history of comorbidity)** | **Full model (No history of comorbidity)** |
| **Variable** | n=636,985 | n=636,985 |
| Annual Visit (0 to 9) | 0.94(0.94,0.94) | 0.89(0.89,0.89) |
| **Sex * race** |  |  |
| Non-Hispanic white*male | ref | ref |
| Non-Hispanic white *female | 2.70(2.53,2.87) | 2.10(1.96,2.24) |
| Non-Hispanic black*male | 1.74(1.71,1.78) | 1.54(1.50,1.57) |
| Non-Hispanic black*female | 5.54(4.58,6.70) | 3.98(3.21,4.93) |
| Hispanic*male | 1.27(1.23,1.30) | 1.09(1.06,1.12) |
| Hispanic*female | 2.69(1.88,3.84) | 1.87(1.29,2.70) |
| Other race*male | 1.12(1.08,1.18) | 0.99(0.95,1.04) |
| Other race*female | 3.08(2.17,4.36) | 2.62(1.80,3.82) |
| **Location of residence** |  |  |
| Urban | ref | ref |
| Rural | 1.28(1.26,1.30) | 1.24(1.22,1.25) |
| Age (per year) | 0.99(0.99,0.99) | 0.99(0.99,0.99) |
| **Marital status** |  |  |
| Married vs unmarried (ref.) | 0.84(0.83,0.85) | 0.94(0.92,0.95) |
| Disability (>50% service-related) | 1.03(1.00,1.04) | 1.00(0.98,1.01) |
| **Smoking Status** |  |  |
| Smoker vs non-smoker (ref.) | 1.11(1.09,1.13) | 1.01(1.00,1.03) |
| Number primary care visits (per year) | 0.99(0.99,0.99) | 0.99(0.99,0.99) |
| **A1C** |  |  |
| A1C >8% (64 mmol/mol) vs A1C ≤8% (64 mmol/mol) (ref) | 1.34(1.32,1.35) | 1.21(1.19,1.22) |
| **ASCVD** |  |  |
| Acute coronary syndr. | 1.10(1.08,1.12) | 0.94(0.92,0.95) |
| Atheroscl. cerebro. dis. | 1.17(1.15,1.19) | 1.06(1.04,1.08) |
| Coronary heart dis. | 0.59(0.58,0.60) | 0.56(0.55,0.57) |
| Peripheral artery dis. | 0.93(0.92,0.93) | 0.90(0.89,0.91) |
| **Statins prescribed** |  |  |
| No statins | ref | ref |
| Low/moderate-intensity statin | 0.34(0.32,0.36) | 0.41(0.38,0.44) |
| High-intensity statin dose | 0.29(0.27,0.31) | 0.29(0.27,0.31) |
| **Dual VA-CMS status** |  |  |
| >80% VA utilization | ref | ref |
| 50-80% VA utilization | 1.00(0.99,1.02) | 0.98(0.96,0.99) |
| < 50% VA utilization | 0.99(0.98,1.00) | 0.93(0.92,0.94) |
| **History of comorbidity** |  |  |
| Psychiatric disorder |  |  |
| Depression |  |  |
| Inter Cluster Correlation | 0.56(0.56,0.56) | 0.56(0.56,0.56) |

**Additional File 5: Sex-Location: Sequential models for the odds of elevated LDL cholesterol (in mg/dL), 2007-2016.**

| **Odds Ratios (95% Confidence Intervals) for multiple imputation of Xtlogit (Logistic Random-Intercept Models)** | | |
| --- | --- | --- |
|  | **LDL≥ 100** | **LDL≥ 70** |
|  | **Full model** | **Full Model** |
| **Variable** | n=636,985 | n=636,985 |
| Annual Visit (0 to 9) | 0.94(0.94,0.94) | 0.89 (0.89,0.89) |
| **Sex * location** |  |  |
| male*urban | ref | ref |
| male*rural | 1.28 (1.26, 1.30) | 1.23 (1.22, 1.25) |
| female*urban | 2.66 (2.48, 2.85) | 2.12 (1.97, 2.28) |
| female*rural | 3.26 (2.94, 3.62) | 2.54 (2.27, 2.83) |
| **Race-ethnicity** |  |  |
| non-Hispanic white | ref | ref |
| non-Hispanic black | 1.76(1.72,1.80) | 1.55(1.51,1.58) |
| Hispanic | 1.25(1.21,1.29) | 1.08(1.05,1.11) |
| Other race | 1.13(1.08,1.19) | 1.00(0.96,1.05) |
| Age (per year) | 0.99 (0.99, 1.00) | 0.99(0.99,0.99) |
| **Marital status** |  |  |
| Married vs unmarried (ref.) | 0.86(0.84,0.87) | 0.94(0.93,0.96) |
| Disability (>50% service-related) | 0.98(0.96,1.00) | 0.98(0.96,1.00) |
| **Smoking Status** |  |  |
| Smoker vs non-smoker (ref.) | 1.10(1.08,1.12) | 1.00(0.99,1.03) |
| Number primary care visits (per year) | 0.99(0.99,0.99) | 0.99(0.99,0.99) |
| **A1C** |  |  |
| A1C >8% (64 mmol/mol) vs A1C ≤8% (64 mmol/mol) (ref) | 1.33(1.32,1.35) | 1.20(1.19,1.22) |
| **ASCVD** |  |  |
| Acute coronary syndr. | 1.09(1.07,1.11) | 0.93(0.92,0.95) |
| Atheroscl. cerebro. dis. | 1.14(1.12,1.16) | 1.05(1.03,1.07) |
| Coronary heart dis. | 0.58(0.58,0.59) | 0.56(0.55,0.57) |
| Peripheral artery dis. | 0.93(0.91,0.94) | 0.89(0.88,0.91) |
| **Statins prescribed** |  |  |
| No statins | ref | ref |
| Low/moderate-intensity statin | 0.34(0.32,0.36) | 0.41(0.38,0.43) |
| High-intensity statin | 0.29(0.27,0.31) | 0.29(0.27,0.31) |
| **Dual VA-CMS status** |  |  |
| >80% VA utilization | ref | ref |
| 50-80% VA utilization | 1.00 (0.98,1.01) | 0.98(0.96,0.99) |
| < 50% VA utilization | 0.99 (0.98,1.00 | 0.93(0.92,0.94) |
| **History of comorbidity** |  |  |
| Psychiatric disorder | 1.16(1.14,1.18) | 1.06(1.04,1.07) |
| Depression | 1.17(1.15,1.19) | 1.08(1.07,1.10) |
| Inter Cluster Correlation | 0.56(0.56,0.57) | 0.56(0.56,0.56) |

**Additional File 6: Sex-Race: Sequential models for the odds of elevated LDL cholesterol (in mg/dL), 2007-2016.**

| **Odds Ratios (95% Confidence Intervals) for multiple imputation of Xtlogit (Logistic Random-Intercept Models)** | | |
| --- | --- | --- |
|  | **LDL≥100** | **LDL≥70** |
|  | **Full model** | **Full model** |
| **Variable** | n=636,985 | n=636,985 |
| Annual Visit (0 to 9) | 0.94(0.94,0.94) | 0.89(0.89,0.89) |
| **Sex * race** |  |  |
| Non-Hispanic white*male | ref | ref |
| Non-Hispanic white *female | 2.60(2.44,2.77) | 2.06(1.93,2.20) |
| Non-Hispanic black*male | 1.76(1.72,1.80) | 1.54(1.51,1.58) |
| Non-Hispanic black*female | 5.38(4.45,6.51) | 3.93(3.18,4.87) |
| Hispanic*male | 1.25(1.21,1.29) | 1.08(1.05,1.11) |
| Hispanic*female | 2.56(1.79,3.66) | 1.83(1.26,2.64) |
| Other race*male | 1.13(1.08,1.19) | 1.00(0.95,1.04) |
| Other race*female | 2.98(2.17,4.22) | 2.58(1.77,3.76) |
| **Location of residence** |  |  |
| Urban | ref | ref |
| Rural | 1.28(1.26,1.30) | 1.23(1.22,1.25) |
| Age (per year) | 0.99(0.99,0.99) | 0.99(0.99,0.99) |
| **Marital status** |  |  |
| Married vs unmarried (ref.) | 0.86(0.84,0.87) | 0.94(0.93,0.96) |
| Disability (>50% service-related) | 0.98(0.96,1.00) | 0.98(0.96,1.00) |
| **Smoking Status** |  |  |
| Smoker vs non-smoker (ref.) | 1.10(1.08,1.12) | 1.01(0.99,1.03) |
| Number primary care visits (per year) | 0.99(0.99,0.99) | 0.99(0.99,0.99) |
| **A1C** |  |  |
| A1C >8% (64 mmol/mol) vs A1C ≤8% (64 mmol/mol) (ref) | 1.33(1.32,1.35) | 1.20(1.19,1.22) |
| **ASCVD** |  |  |
| Acute coronary syndr. | 1.09(1.07,1.12) | 0.93(0.92,0.95) |
| Atheroscl. cerebro. dis. | 1.14(1.12,1.16) | 1.05(1.03,1.07) |
| Coronary heart dis. | 0.58(0.58,0.59) | 0.56(0.55,0.57) |
| Peripheral artery dis. | 0.93(0.91,0.94) | 0.89(0.89,0.91) |
| **Statins prescribed** |  |  |
| No statins | ref | ref |
| Low/moderate-intensity statin | 0.34(0.32,0.36) | 0.41(0.38,0.43) |
| High-intensity statin | 0.29(0.27,0.31) | 0.29(0.27,0.31) |
| **Dual VA-CMS status** |  |  |
| >80% VA utilization | ref | ref |
| 50-80% VA utilization | 1.00(0.98,1.01) | 0.98(0.96,0.99) |
| < 50% VA utilization | 0.99(0.98,1.00) | 0.93(0.92,0.94) |
| **History of comorbidity** |  |  |
| Psychiatric disorder | 1.16(1.14,1.18) | 1.06(1.04,1.07) |
| Depression | 1.17(1.15,1.19) | 1.08(1.07,1.10) |
| Inter Cluster Correlation | 0.56(0.56,0.56) | 0.56(0.56,0.56) |

**Additional File 7: GEE Model for the odds of LDL missingness**

| **Predictor variables** | **Odds Ratios (95% CI) for missingness** |
| --- | --- |
|  | **LDL** |
| **Statin use** |  |
| Low vs none (ref.) | 0.002(0.002,0.002) |
| High vs none (ref.) | 0.002(0.002,0.002) |
| **Dual VA-CMS utilization (%)** |  |
| Dual 50-80% vs Dual >80% (ref.) | 1.00(0.98,1.02) |
| Dual <50% vs Dual >80% (ref.) | 1.02(1.00,1.03) |
| **Hemoglobin A1C >8%** | 1.20(1.18,1.22) |
| Primary care visit | 0.99(0.99,0.99) |
| **Annual visit** | 0.91(0.91,0.91) |
| **Age** | 1.00(1.00,1.00) |
| **Race/ethnicity** |  |
| NHB vs NHW (ref.) | 1.00(0.98,1.04) |
| Hispanic vs NHW(ref.) | 1.16(1.12,1.21) |
| Other race vs NHW (ref.) | 0.83(0.78,0.88) |
| Female vs male (ref.) | 1.00(0.93,1.07) |
| Disability >50% service-related vs <50% | 0.98(0.96,1.00) |
| Rural vs Urban (ref.) | 0.95(0.0.93,0.96) |
| Smoker vs nonsmoker (ref.) | 1.00(0.99,1.03) |
| Married vs nonmarried (ref.) | 0.95(0.93,0.97) |
| MACE_ACS Yes vs No (ref.) | 1.00(0.98,1.02) |
| MACE_ACD Yes vs No (ref.) | 1.01(0.99,1.04) |
| MACE_CHD Yes vs No (ref.) | 1.10(1.08,1.12) |
| MACE_PAD Yes vs No (ref.) | 1.03(1.00,1.04) |
| Elixhauser comorbidities (sum) | 1.02(1.00,1.04) |
| CHF Yes vs No (ref.) | 1.02(1.00,1.05) |
| VALVE Yes vs No (ref.) | 0.95(0.93,0.99) |
| PULMCIRC Yes vs No (ref.) | 1.00(0.97,1.03) |
| HTN Yes vs No (ref.) | 0.95(0.92,0.98) |
| HTNCX Yes vs No (ref.) | 0.96(0.93,0.99) |
| PARA Yes vs No (ref.) | 0.95(0.92,0.98) |
| NEURO Yes vs No (ref.) | 1.00(0.98,1.03) |
| CHRNLUNG Yes vs No (ref.) | 0.99(0.97,1.02) |
| HYPOTHY Yes vs No (ref.) | 0.99(0.97,1.02) |
| RENLFAIL Yes vs No (ref.) | 1.07(1.04,1.10) |
| LIVER Yes vs No (ref.) | 0.98(0.94,1.02) |
| ULCER Yes vs No (ref.) | 1.18(1.08,1.29) |
| AIDS Yes vs No (ref.) | 1.07(0.88,1.31) |
| LYMPH Yes vs No (ref.) | 1.02(0.97,1.08) |
| METS Yes vs No (ref.) | 1.00(0.96,1.03) |
| TUMOR Yes vs No (ref.) | 1.02(0.99,1.04) |
| ARTH Yes vs No (ref.) | 0.99(0.96,1.03) |
| COAG Yes vs No (ref.) | 0.98(0.95,1.00) |
| OBESE Yes vs No (ref.) | 1.02(1.00,1.05) |
| WGHTLOSS Yes vs No (ref.) | 0.98(0.95,1.00) |
| LYTES Yes vs No (ref.) | 1.03(1.00,1.05) |
| BLDLOSS Yes vs No (ref.) | 0.98(0.95,1.02) |
| ANEMDEF Yes vs No (ref.) | 0.95(0.92,0.97) |
| ALCOHOL Yes vs No (ref.) | 1.03(1.00,1.07) |
| DRUG Yes vs No (ref.) | 1.07(1.02,1.13) |
| PSYCH Yes vs No (ref.) | 1.09(1.06,1.12) |
| DEPRESS Yes vs No (ref.) | 1.02(0.99,1.04) |

**Additional File 8: Demographic and clinical characteristics by location of residence, 2007-2016**

|  |  | **Location** | | |
| --- | --- | --- | --- | --- |
| **Variable** | **Level** | **Urban** | **Rural** | **Total** |
| n | No. (%) | 462,676  (64.8%) | 251,536  (35.2%) | 714,212 (100.0%) |
| Age | mean (std) | 76.2 (6.3) | 75.5 (6.1) | 75.9(6.2) |
| Sex | Male (%) | 98.5 | 98.9 | 98.7 |
| Mortality rate | (year 2007) | 67.7 | 67.8 | 67.8 |
| Race-ethnicity | non-Hispanic white (%) | 77.9 | 91.6 | 82.7 |
|  | non-Hispanic black (%) | 12.8 | 4.9 | 10.0 |
|  | Hispanic (%) | 7.0 | 1.7 | 5.2 |
|  | Other race (%) | 2.3 | 1.8 | 2.1 |
| Marital status | Married (%) | 58.3 | 62.2 | 59.6 |
| Disability | >50% service-related (%) | 17.4 | 15.9 | 16.9 |
| Location of residence | Rural (%) | -- | -- | -- |
| Smoking status | Smoker (%) | 13.3 | 15.4 | 14.1 |
| Number of Elixhauser comorbidities | mean number per group (std) | 7.9 (3.3) | 8.3 (3.3) | 8.1 (3.3) |
| Number of primary care visits | mean per year (std) | 4.7 (4.1) | 4.6 (4.0) | 4.7 (4.1) |
| Hemoglobin A1c | Percent ≥ 8%  (64 mmol/mol) | 10.1 | 10.2 | 10.1 |
|  | Percent<8%  (64 mmol/mol) | 62.7 | 63.0 | 62.8 |
|  | Missing | 27.2 | 26.8 | 27.1 |
| ASCVD | Acute coronary syndrome | 23.1 | 26.5 | 24.3 |
|  | Atherosclerotic cerebrovasc. dis. | 20.0 | 21.9 | 20.7 |
|  | Coronary heart disease | 64.5 | 69.1 | 66.1 |
|  | Peripheral artery disease | 45.3 | 48.0 | 46.2 |
| Statin use (%) | None | 18.4 | 17.7 | 18.1 |
|  | Low | 70.5 | 72.3 | 71.2 |
|  | High | 3.6 | 3.5 | 3.5 |
|  | Missing | 7.5 | 6.5 | 7.2 |
| Dual VA-CMS utilization (%) | > 80% VA utilization | 48.7 | 44.5 | 47.2 |
|  | 50%-80% VA utilization | 7.1 | 7.7 | 7.3 |
|  | < 50% VA utilization | 31.5 | 34.9 | 32.7 |
|  | Missing | 12.7 | 12.9 | 12.8 |
| History of comorbidity (%) | Psychiatric disorder | 18.8 | 19.2 | 19.0 |
|  | Depression | 30.6 | 32.0 | 31.1 |
| LDL≥ 70 (%) | No | 18.9 | 18.6 | 18.8 |
|  | Yes | 51.3 | 54.4 | 52.4 |
|  | Missing | 29.8 | 27.0 | 28.8 |
| LDL≥ 100 (%) | No | 50.8 | 51.6 | 51.1 |
|  | Yes | 19.4 | 21.4 | 20.1 |
|  | Missing | 29.8 | 27.0 | 28.8 |
| All values are at visit of baseline year (2007) | | | | |

**Additional File 9: Demographic and clinical characteristics by race/ethnicity status, 2007-2016**

| **Variable** | **Level** | **Race/ethnicity** | | | | **Total** |
| --- | --- | --- | --- | --- | --- | --- |
|  |  | **NHW** | **NHB** | **Hispanic** | **Other** |  |
| n | No. (%) | 590,872 (82.7%) | 71,275 (10.0%) | 36,790 (5.2%) | 15,275 (2.1%) | 714,212 (100%) |
| Age | mean (std) | 76.1 (6.2) | 74.9 (6.2) | 75.6 (6.1) | 75.3 (6.4) | 75.9 (6.3) |
| Mortality rate | (in 2007) | 68.9 | 64.1 | 59.7 | 62.5 | 67.8 |
| Sex | Male (%) | 98.6 | 98.7 | 99.3 | 98.1 | 98.7 |
| Marital status | Married (%) | 61.1 | 46.5 | 60.6 | 61.9 | 59.6 |
| Disability | >50% service-related (%) | 15.7 | 23.3 | 18.9 | 29.9 | 16.9 |
| Location of residence | Rural (%) | 39.0 | 17.1 | 11.9 | 30.1 | 35.2 |
| Smoking status | Smoker (%) | 13.7 | 17.6 | 13.3 | 14.1 | 14.1 |
| Number of Elixhauser comorbidities | mean number per group (std) | 8.1 (3.3) | 8.3 (3.2) | 7.4 (3.2) | 7.6 (3.3) | 8.1 (3.3) |
| Number of primary care visits | mean per year (std) | 4.7 (4.1) | 4.3 (3.7) | 4.6 (3.9) | 4.6 (4.0) | 4.7 (4.1) |
| Hemoglobin A1c | percent ≥ 8%  (64 mmol/mol) | 9.3 | 14.2 | 14.5 | 10.7 | 10.1 |
|  | percent < 8%  (64 mmol/mol) | 63.9 | 59.1 | 54.9 | 59.5 | 62.8 |
|  | Missing | 26.8 | 26.7 | 30.6 | 29.8 | 27.1 |
| ASCVD (%) | Acute coronary syndrome | 24.7 | 21.8 | 23.7 | 22.3 | 24.3 |
|  | Atherosclerotic cerebrovasc. dis. | 20.6 | 22.0 | 19.2 | 18.7 | 20.6 |
|  | Coronary heart disease | 68.2 | 56.1 | 53.7 | 60.6 | 66.1 |
|  | Peripheral artery disease | 46.7 | 45.8 | 42.6 | 41.2 | 46.2 |
| Statin use (%) | None | 18.1 | 18.7 | 16.4 | 19.2 | 18.1 |
|  | Low | 71.3 | 69.0 | 74.4 | 67.2 | 71.2 |
|  | High | 3.6 | 3.6 | 2.4 | 3.3 | 3.5 |
|  | Missing | 7.0 | 8.7 | 6.8 | 10.3 | 7.2 |
| Dual VA-CMS utilization (%) | > 80% VA utilization | 44.9 | 56.8 | 65.0 | 48.9 | 47.2 |
|  | 50%-80% VA utilization | 7.5 | 6.4 | 6.2 | 7.1 | 7.3 |
|  | < 50% VA utilization | 35.5 | 19.7 | 14.9 | 27.9 | 32.7 |
|  | Missing | 12.1 | 17.1 | 13.9 | 16.1 | 12.8 |
| History of comorbidity (%) | Psychiatric disorder | 18.5 | 20.7 | 22.6 | 18.7 | 19.0 |
|  | Depression | 31.4 | 27.5 | 33.5 | 29.4 | 31.1 |
| LDL≥ 70 (%) | No | 19.4 | 14.6 | 17.0 | 18.1 | 18.8 |
|  | Yes | 52.0 | 55.1 | 54.8 | 50.8 | 52.4 |
|  | Missing | 28.6 | 30.3 | 28.2 | 31.1 | 28.8 |
| LDL≥ 100 (%) | No | 52.1 | 44.6 | 48.3 | 48.6 | 51.1 |
|  | Yes | 19.3 | 25.2 | 23.5 | 20.3 | 20.1 |
|  | Missing | 28.6 | 30.2 | 28.2 | 31.1 | 28.8 |
| Note: All values are computed at the visit of baseline year (2007) | | | | | | |
